# Transcranial Doppler Monitoring of Intracranial Pressure Plateau Waves

**DOI:** 10.1007/s12028-016-0356-5

**Published:** 2016-12-20

**Authors:** Danilo Cardim, Bernhard Schmidt, Chiara Robba, Joseph Donnelly, Corina Puppo, Marek Czosnyka, Peter Smielewski

**Affiliations:** 1 Brain Physics Laboratory, Division of Neurosurgery, Department of Clinical Neurosciences, University of Cambridge, Cambridge, UK; 2 Department of Neurology, University Hospital Chemnitz, Chemnitz, Germany; 3 Neurosciences Critical Care Unit, Addenbrooke’s Hospital, Cambridge University NHS Foundation Trust, Cambridge, UK; 4 School of Medicine Hospital, Universidad de la República, Montevideo, Uruguay; 5 Institute of Electronic Systems, Warsaw University of Technology, Warsaw, Poland

**Keywords:** Transcranial Doppler, Noninvasive ICP, Traumatic brain injury, ICP plateau waves

## Abstract

**Background:**

Transcranial Doppler (TCD) has been used to estimate ICP noninvasively (nICP); however, its accuracy varies depending on different types of intracranial hypertension. Given the high specificity of TCD to detect cerebrovascular events, this study aimed to compare four TCD-based nICP methods during plateau waves of ICP.

**Methods:**

A total of 36 plateau waves were identified in 27 patients (traumatic brain injury) with TCD, ICP, and ABP simultaneous recordings. The nICP methods were based on: (1) interaction between flow velocity (FV) and ABP using a “black-box” mathematical model (*nICP_BB*); (2) diastolic FV (*nICP_FV*_*d*_); (3) critical closing pressure (*nICP_CrCP*), and (4) pulsatility index (*nICP_PI*). Analyses focused on relative changes in time domain between ICP and noninvasive estimators during plateau waves and the magnitude of changes (*∆* between baseline and plateau) in real ICP and its estimators. A ROC analysis for an ICP threshold of 35 mmHg was performed.

**Results:**

In time domain, *nICP_PI*, *nICP_BB*, and *nICP_CrCP* presented similar correlations: 0.80 ± 0.24, 0.78 ± 0.15, and 0.78 ± 0.30, respectively. *nICP_FV*_*d*_ presented a weaker correlation (*R* = 0.62 ± 0.46). Correlations between ∆ICP and ∆nICP were better represented by *nICP_CrCP and BB*, *R* = 0.48, 0.44 (*p* < 0.05), respectively. *nICP_FV*_*d*_ *and PI* presented nonsignificant *∆* correlations. ROC analysis showed moderate to good areas under the curve for all methods: *nICP_BB*, 0.82; *nICP_FV*_*d*_, 0.77; *nICP_CrCP*, 0.79; and *nICP_PI*, 0.81.

**Conclusions:**

Changes of ICP in time domain during plateau waves were replicated by nICP methods with strong correlations. In addition, the methods presented high performance for detection of intracranial hypertension. However, absolute accuracy for noninvasive ICP assessment using TCD is still low and requires further improvement.

## Introduction

Acute intracranial hypertension (ICH) is a recurrent cause of secondary injury in patients under neurocritical care. Intracranial pressure (ICP) represents an essential monitoring modality in the correct clinical management of several neurological diseases carrying risk of potentially lethal ICH. ICP is a complex variable, consisting of four components modulated by different physiological mechanisms: (1) inflow and volume of arterial blood, (2) venous blood outflow, (3) cerebrospinal fluid (CSF) circulation, and (4) brain parenchymal or lesion volume. The importance of ICP is not always associated with its absolute value, but with monitoring of its dynamics in time and with identifying which of the above mentioned components are responsible for the observed pattern of ICH [1]. This is essential, as every component that elevates ICP requires different countermeasures (like short-term hyperventilation to control 1; head elevation to control 2; extra ventricular drainage to control 3; osmotherapy or craniectomy to control component 4).

First described by Janny [2] in 1950 and later by Lundberg [3], plateau waves of ICP (or Lundberg A waves) [3] are characterized by sudden and relevant increases in ICP (generally above 40 mmHg [4]), related to increased volume of arterial blood in response to arterial vasodilation stimulus. Such phenomena may develop in patients presenting intact cerebral autoregulation [4] and low cerebrospinal compensatory reserve [5], suffering from a wide range of cerebral pathological conditions including traumatic brain injury (TBI) [4], idiopathic intracranial hypertension [6], subarachnoid hemorrhage [7], brain tumors, hydrocephalus [8], and craniosynostosis [9].

During the occurrence of plateau waves, ICP increases from normal or slightly elevated to uncompensated, acute ICH, with relatively stable arterial blood pressure (ABP) [10]. The mechanism driving plateau waves can be described as a “vasodilatory cascade,” initiated by a vasodilatory stimulation (reduction in ABP during intact autoregulation, for instance) [4]. Following this, a rapid increase in cerebral blood volume (CBV) leads to a cyclic rise in ICP, decrease in cerebral perfusion pressure (CPP), further vasodilation and further rise of ICP. This cycle is maintained until the cerebral vasculature reaches a state of maximum vasodilation [10]. The reverse of this positive feedback loop occurs when a vasoconstrictive stimulus initiates a vasoconstrictive cascade, decreasing CBV with a consequent drop in ICP toward normal levels [10, 11].

Transcranial Doppler ultrasonography (TCD), owing to its specificity to detect changes in cerebrovascular dynamics [12], offers non-quantitative measurements of cerebral blood flow (CBF). CBF is the blood supply to the brain in a given period of time, and global changes in this parameter can be monitored continuously and noninvasively using TCD-derived cerebral blood flow velocity (FV) [13]. CBV and CBF are usually correlated, as CBV equals CBF multiplied by the cerebral vascular mean transit time [14]. Some studies have demonstrated that specific patterns of FV waveform reflect inadequate cerebral perfusion caused by a decrease in CPP [15, 16] (as it occurs during plateau waves, for instance). In such cases, ICP increases lead to decreases in CPP by closing arterioles at higher diastolic pressures, thus causing remarkable drops in diastolic flow velocity (FV_d_). Consequently, there is a generalized decrease in mean flow velocity (FV_m_). These characteristics observed in the FV waveform pattern can be used as indicators of cerebral perfusion derangements and have been applied as variables for noninvasive ICP estimations [17]. However, the inherent difficulty of ICP estimation in such a scenario is the lack of reliable individual calibration of estimators in appropriate units (mmHg).

Although it has been shown that different TCD-based nICP estimators present with different measures of accuracy [18], there are reports suggesting that these methods are more accurate when changes of ICP are related to vasogenic phenomena [19, 20]. Considering these assumptions, the aim of this study was to assess a set of four previously described TCD-based nICP methods in a cohort of patients who presented changes in ICP purely of vasogenic origin (specifically plateau wave increases of ICP) to verify whether these methods reliably predict ICP under such conditions.

## Methods

### Patients

From a database of 446 adult TBI patients [median age of patients: 30 years (interquartile range (IQR) 18–78, with 75% being male), with simultaneous recordings of ABP, ICP, and TCD, 27 patients were identified where at least one plateau wave occurred during the monitoring period. Plateau waves were identified as sudden and spontaneous increases in ICP (and pulse amplitude of ICP), during which CPP and FV_m_ dropped with a relatively stable ABP. A total of 36 plateau waves were identified during the TCD monitoring and retrospectively analyzed for the purpose of this study. The mean length of plateau waves was 13 min with a range of 2–30 min. TBI data were recorded during routine clinical TCD investigations of cerebral autoregulation on the Neurosciences Critical Care Unit at Addenbrooke’s Hospital, Cambridge UK (included in Protocol 30 REC 97/290), and at the Universidad de la República School of Medicine Hospital, Montevideo, Uruguay (one recording, with approval of the local ethical committee). The retrospective analysis was performed as part of an anonymous clinical audit.

### Monitoring and Data Analysis

ABP was invasively monitored from the radial artery with a pressure monitoring kit (Baxter Healthcare CA, USA; Sidcup, UK), zeroed at the level of the heart. ICP was monitored using an intraparenchymal probe (Codman&Shurtleff or Camino), and FV was monitored exclusively from the M1 segment of the middle cerebral artery through the temporal window with a 2-MHz probe and the Doppler Box (DWL Compumedics) or Neuroguard (Medasonic) TCD devices. The probe was held in place during the entire recording using a head band or frame provided by the TCD device manufacturer. TCD monitoring was ordered routinely in every patient after TBI to assess the state of cerebral autoregulation. The TCD recordings with simultaneous ABP and ICP were performed for periods of 10 min to 1 h by MC, PS and CP. ICP management was not stopped during the TCD recordings, but occasional artifacts (such as ABP flushes and ICP increases generated by suctions) were cleaned out.

Raw signals were digitized using an analog–digital converter (DT2814, Data Translation) sampled at a frequency of 50 Hz and recorded using in-house designed software (separate in Cambridge and Montevideo) and later retrospectively analysed using ICM+ software (Cambridge Enterprise, http://www.neurosurg.cam.ac.uk/icmplus/). The recorded signals were subjected to manual artifacts removal.

All the calculations, including mean values of ABP, ICP, FV, were performed over a 10-s long-sliding window. The minimal and maximum values of FV from every 2-s period were calculated and treated as end diastolic and peak systolic (FV_s_) components, respectively. These components were then averaged over 10 s to give the mean values for FV_d_ and FV_s_.

### Noninvasive ICP Methods

1. Schmidt et al. “black-box” (BB) model [21] (**nICP_BB**):

It is described in terms of a transfer function between ABP and ICP [22, 23], controlled by TCD- and ABP-derived parameters. The rules of this TCD-based linear control had been formerly determined using a multiple regression model between TCD parameters and ABP-ICP transfer function on datasets of reference patients (N = 140, TBI). The model provides continuous full waveform of nICP (in mmHg).

2. Czosnyka et al. [16] (**nICP_FV**_**d**_):

It applies the diastolic flow velocity waveform analysis for the estimation of nCPP (noninvasive CPP). nICP can be calculated as the difference between mean ABP (ABPm) and nCPP (nICP = ABPm − nCPP).

1 $${\text{nICP}} = {\text{ABPm }} \times \left( {1 - \frac{\text{FVd}}{\text{FVm}}} \right) - 14 {\text{mmHg}}$$

14 mmHg is the zero compensation factor established in a cohort of TBI patients [16].

3) Varsos et al. [24] (**nICP_CrCP**):

Similarly, it calculates nICP based on nCPP, in this case specifically using the concept of critical closing pressure (CrCP) [24].

2 $${\text{nCPP}} = {\text{ABPm}} \times \left[ {0.734 - \frac{0.266}{{\sqrt {\left( {{\text{CVR}} \times {\text{Ca}} \times {\text{HR}} \times 2\uppi} \right)^{2} + 1} }}} \right] - 7.026\;{\text{mmHg}}$$

3 $${\text{CVR}} = \frac{\text{ABP}}{\text{FV}}$$

4 $${\text{Ca}} = \frac{\text{CaBV Amp}}{\text{ABP Amp}}$$

CVR (mmHg/(cm/s)) represents cerebral vascular resistance, Ca (cm/mmHg) denotes the compliance of the cerebral arterial bed assuming a non-pulsatile venous blood outflow [25], and HR expresses heart rate (beats/s), with ABP and FV as the required measurements for the calculation of these parameters. CaBV Amp represents the fundamental amplitude of the cerebral arterial blood volume, calculated from FV waveform using a model of non-pulsatile cerebral blood outflow [26]. ABP Amp represents the fundamental amplitude of arterial blood pressure. Finally, nICP can be obtained as the difference between ABP and nCPP (nICP = ABP − nCPP). Constant coefficients (0.734, 0.266, 7.026 mmHg) are derived from analysis of database of retrospective cases (*N* = 232, TBI) [24].

### nICP_PI

Pulsatility index (PI) describes changes in the morphology of the FV waveform. It is a relationship between the difference of FV_s_ and FV_d_ divided by FV_m_ (Eq. 5). The estimation formula for nICP_PI originated from the linear regression between ICP and PI from a population cohort of 292 TBI patients [27] (Eq. 6).

5 $${\text{PI}} = \frac{{{\text{FV}}_{\text{s}} - {\text{FV}}_{\text{d}} }}{{{\text{FV}}_{\text{m}} }}$$

6 $${\text{nICP}} = 4.47 \times {\text{PI }} + 12.68 {\text{mmHg}}$$

### Statistical Analysis

Statistical analysis of the data was conducted with R Studio software (R version 3.1.2). Data were tested for normal distribution using the Shapiro–Wilk test. All plateau waves were treated as independent phenomena. The analysis included Spearman correlations between ∆ICP and ∆nICP (*∆* correlations) and averaged correlations for variations of nICP across time during the course of plateau waves. “*∆*” (magnitude) represents the difference between plateau (at the top of plateau waves) and baseline (before the onset of plateau waves) mean values in each recording. R symbolizes the Spearman correlation coefficient, with the level of significance set at 0.05.

The area under the curve (AUC) of the receiver operating characteristic curve (ROC) was performed to determine the ability of the noninvasive methods to detect raised ICP during plateau waves (using a threshold of 35 mmHg). This threshold was chosen considering the high values of ICP observed during both phases of plateau waves. In this case, 35 mmHg represents mathematically half way between baseline and plateau phases ICP values. The predicting ability is considered reasonable when the AUC is higher than 0.7 and strong when the AUC exceeds 0.8 [28]. Statistical differences between ROC curves were verified using the DeLong’s test for two correlated ROC curves (R package pROC [29]).

## Results

In 27 patients, a total of 36 plateau waves were identified (7 patients presented two plateau waves, and one patient presented 3 plateau waves). Table 1 presents correlations (∆*R*) between ∆ICP and ∆nICP and averaged correlations in time domain.

**Table 1:** Median values (IQR) of the differences between plateau and baseline phase (*∆* [mmHg]), *∆* correlations with ICP, correlations in time domain for all assessed methods and area under ROC curve for detection of ICP above 35 mmHg

Method	*∆*	*R* (∆ICP vs. ∆nICP)	*R* (time domain)	AUC (95% CI)
nICP_BB	9.00 (13.18–5.05)	0.44*	0.78 ± 0.15	0.82 (0.71–0.93)
nICP_FVd	10.10 (17.74–4.56)	0.19	0.62 ± 0.46	0.77 (0.65–0.88)
nICP_CrCP	2.89 (4.12–2.11)	0.48*	0.78 ± 0.30	0.79 (0.67–0.91)
nICP_PI	2.82 (5.20–1.92)	0.30	0.80 ± 0.24	0.81 (0.70–0.91)
ICP	24.49 (26.72–21.19)	–	–	

∆ICP and ∆nICP distributions are significantly different in all cases. AUC prediction estimators for each nICP method considering the threshold of 35 mmHg. *ICP* intracranial pressure, *nICP_BB* estimator based on a black-box mathematical model, *nICP_FVd* estimator based on the diastolic cerebral blood flow velocity, *nICP_CrCP* estimator based on the concept of critical closing pressure, and *nICP_PI* estimator based on the pulsatility index

* *Spearman* correlation coefficient is significant at the 0.05 level

**Fig. 1:**
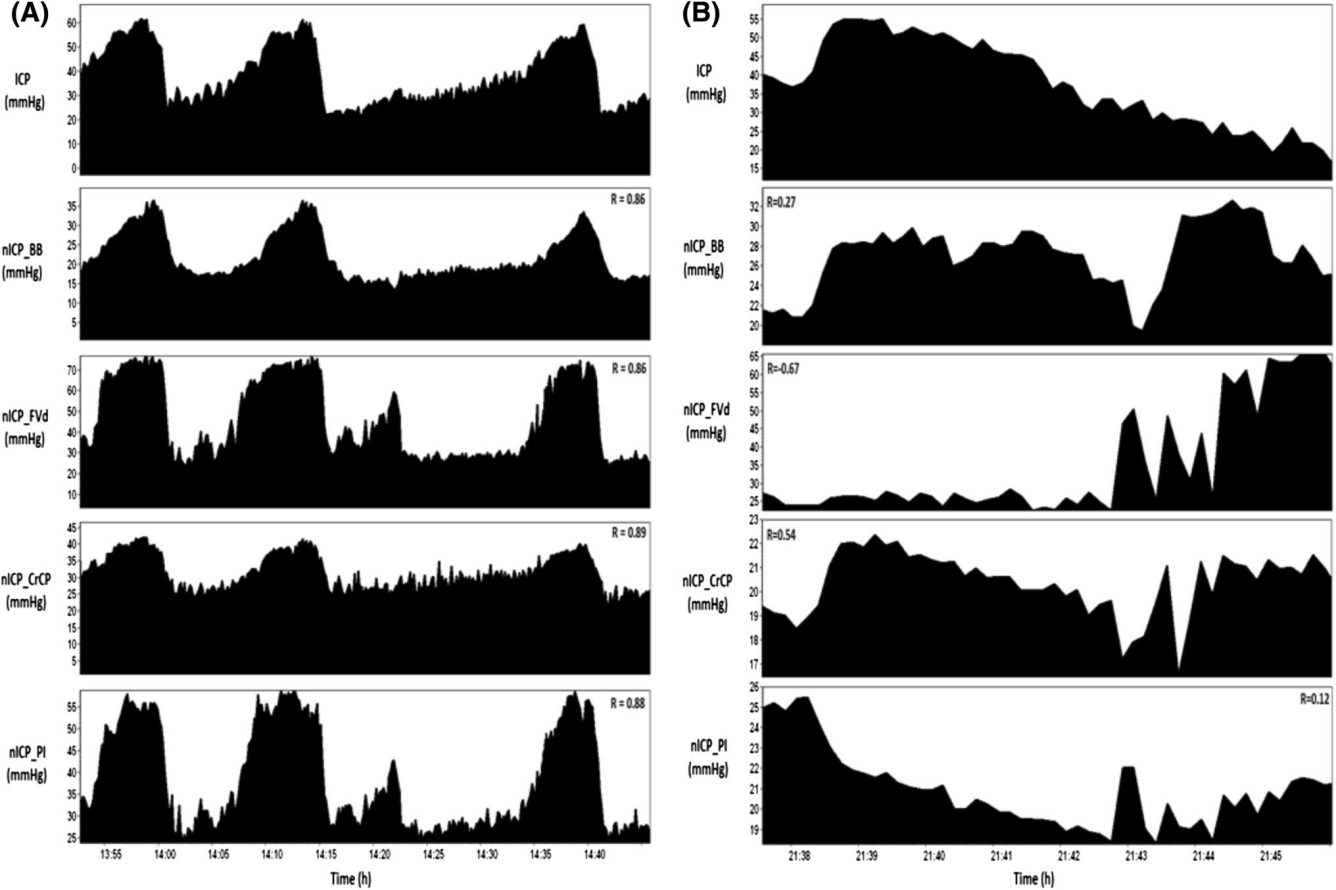
Example of recordings showing reliable and unreliable replications of a plateau waves of ICP by TCD-based nICP methods (panels **a** and **b**, respectively). On Y axis, mean absolute values of ICP and nICPs are presented; and on X axis, relative changes of ICP and nICPs in time domain

Correlations in time domain are independent of mean values of ICP or nICP and represent the ability of a nICP method to replicate relative changes observed in direct ICP across time. In average, correlations in time were reasonably good, with *R* > 0.60 for all methods. nICP_PI, nICP_BB, and nICP_CrCP presented similar averaged correlations in time (*R* ≥ 0.78). Examples of good and poor correlations between ICP and nICP during plateau waves are shown in Fig. 1.

**Fig. 2:**
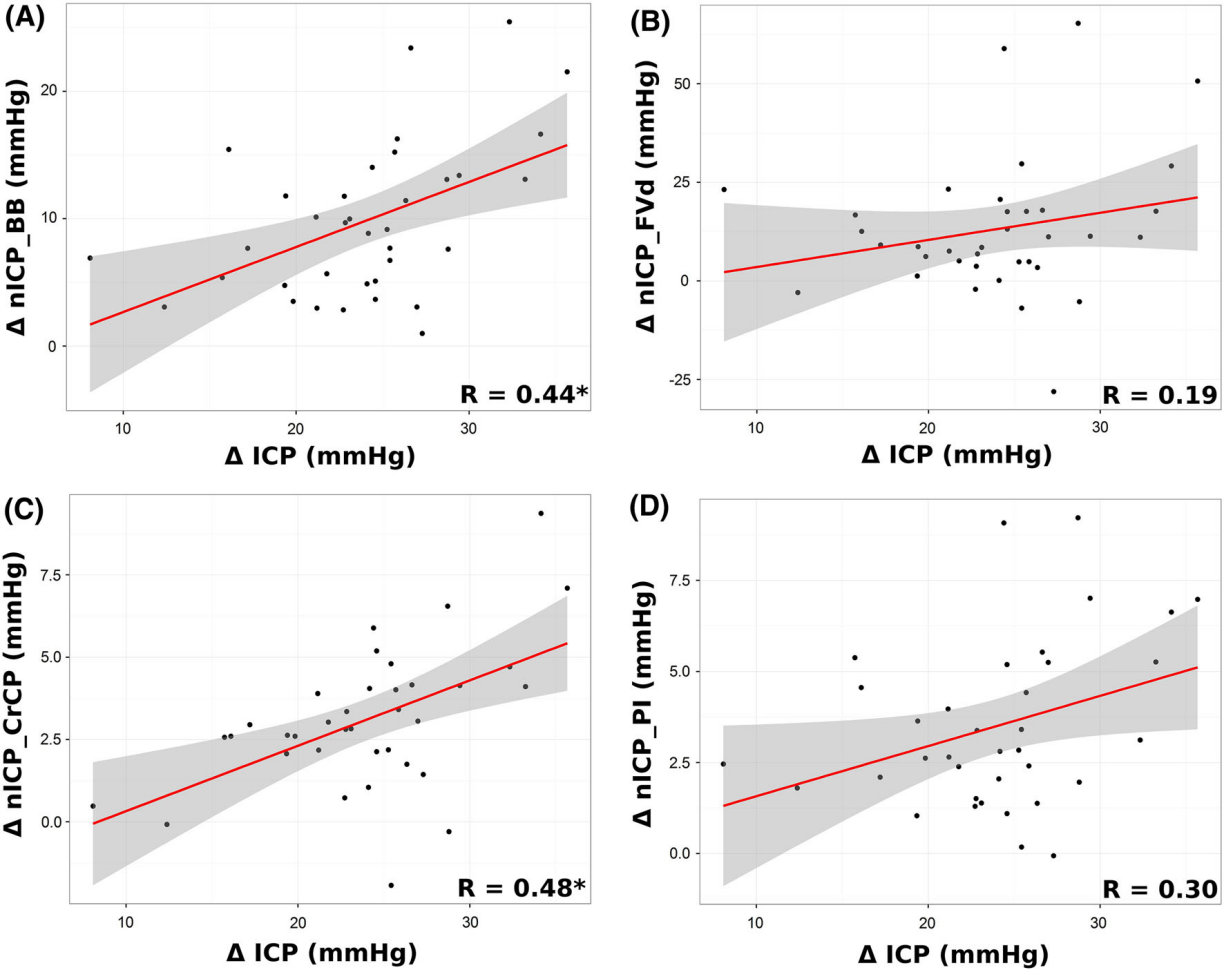
Correlations plot between ∆ICP and ∆nICP for all methods. (*asterisk*) Spearman correlation coefficient is significant at the 0.05 level

Correlations between ∆ICP and ∆nICP were better represented by nICP_CrCP and nICP_BB, while the other methods presented inferior and nonsignificant correlations (Fig. 2). All ∆nICP mean values were significantly underestimated in comparison with ∆ICP.

Table 1 also contains the prediction values for each nICP method according to the ROC analysis. The best AUC value for predicting intracranial hypertension (i.e., ICP ≥ 35 mmHg) was presented by nICP_PI. Nevertheless, all methods presented AUC above 0.7, an indication of reasonable prediction abilities for detecting ICH. The DeLong’s test for two correlated ROC curves did not reveal any statistically significant difference between nICP methods. The ICP thresholds yielding the best sensitivity and specificity were 27.41 mmHg (AUC = 0.86), 28.42 mmHg (AUC = 0.79), 23.08 mmHg (AUC = 0.75), and 20.28 mmHg (AUC = 0.84), respectively, for nICP_BB, FVd, CrCP, and PI, respectively.

nICP_CrCP and nICP_PI presented null values for specificity and negative predictive value, indicating that values of these two estimators during plateau waves were always below 35 mmHg (although they reacted to rise in ICP, a remarkable underestimation of real pressure was observed). Nevertheless, all methods presented good positive predictive values (65, 69, 58, and 58% for nICP_BB, FVd, CrCP, and PI, respectively), which otherwise would indicate that many of the positive results were false positives.

**Fig. 3:**
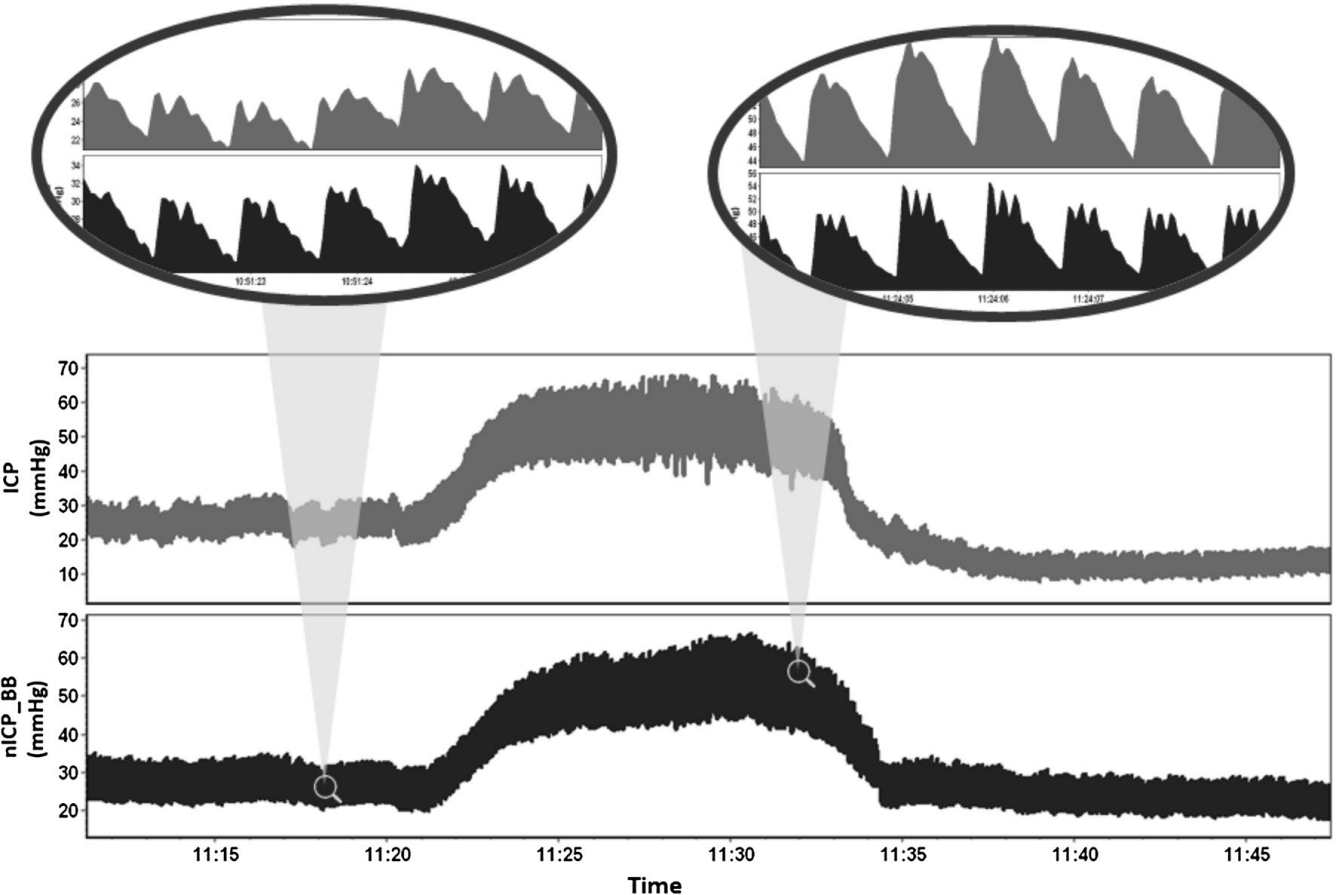
Example of plateau wave recording with direct ICP and simulated ICP (nICP_BB). In comparison with baseline phase, at the top of plateau waves nICP_BB presented an increased pulse amplitude, in agreement with ICP. The characteristic triangular shape of ICP waveform with three distinctive peaks observed during plateau waves was replicated by nICP_BB

Table 2 presents the median values (IQR) for the physiological variables evaluated during baseline and plateau phases. During plateau waves, ICP (and ICP pulse amplitude—ICP Amp) increased and CPP decreased significantly, whereas ABP remained unchanged. FV_m_ and FV_d_ presented significant decreases, whereas FV_s_ increased significantly. This resulted in significant increase in PI and decrease in CVR. As the only nICP method provides ICP waveform, nICP_BB presented increased pulse amplitude (nICP_BB Amp), even with replication of the characteristic triangular waveform shape observed during plateau waves [30] (Fig. 3). The correlation between ∆ICP Amp and ∆nICP_BB Amp was significant (*R* = 0.50, *p* < 0.05); however, nICP_BB Amp was significantly underestimated in comparison with ICP Amp during plateau phase (Bias = −2.88 mmHg, *p* < 0.05).

**Table 2:** Median (IQR) values for all physiological variables estimated during baseline and plateau, with their respective ∆ correlations with ∆ICP and ∆ABP

Variable	Baseline	Plateau
ICP	22.81 (28.49–19.91)	46.45 (55.22–40.64)*
ABP	93.34 (98.30–86.14)	93.55 (97.44–85.28)
HR	75.72 (84.91–65.42)	74.73 (80.45–66.14)*
CPP	70.89 (78.22–60.38)	45.91 (52.87–37.17)*
FV_m_	57.31 (71.87–43.16)	45.52 (63.96–33.15)*
FV_s_	109.35 (132.35–92.78)	113.55 (141.72–99.44)*
FV_d_	31.04 (40.24–23.26)	22.99 (36.60–10.97)*
PI	1.39 (1.68–1.21)	2.00 (2.83–1.68)*
CVR	1.23 (1.47–1.00)	0.99 (1.25–0.68)*
CBV Amp	2.38 (3.73–1.82)	3.36 (4.77–2.42)*
CrCP	51.37 (56.88–41.89)	65.33 (70.67–52.07)*
ICP Amp	2.21 (2.58–1.70)	6.16 (7.88–4.90)*
nICP_BB Amp	1.97 (2.70–1.61)	3.44 (4.07–2.67)*

*ABP* arterial blood pressure (mmHg), *CBV Amp* cerebral blood volume amplitude (mmHg), *CPP* cerebral perfusion pressure (mmHg), *CrCP* critical closing pressure (mmHg), *CVR* cerebral vascular resistance (mmHg*s/cm), *FV* cerebral blood flow velocity (m, mean; s, systolic; d, diastolic) (cm/s), *HR* heart rate (beats/min), *ICP Amp* pulse amplitude of ICP (mmHg), *nICP_BB Amp* amplitude of non-invasive ICP based on the black box model, *PI* pulsatility index

* At the 0.05 level, distributions between baseline and plateau are significantly different

## Discussion

In this work, we present the results of an assessment of a set of TCD-based nICP methods in a cohort of patients with changes in ICP purely of vasogenic origin (i.e., plateau waves of ICP). Within the evaluated methods, only nICP_BB and nICP_CrCP presented significant correlations with ∆ICP. On the other hand, changes in ICP recorded in time  were confidently replicated by all methods.

Changes of ICP in time domain have been assessed in other clinical conditions, and it appears to be highly accurate when changes in ICP are of vasogenic origin. For instance, during CSF infusion tests a controlled and artificial increase in CSF circulation causes an elevation in ICP similar to the pattern observed during plateau waves. Nevertheless, the correlations in time domain obtained with the same nICP methods were considerably weaker [19] in comparison with those observed here, where ICP changes were specifically related to increases in cerebral arterial blood volume. Moreover, such findings were not better replicated when another TBI cohort was analyzed [31], in which changes in ICP across time were not always related to vasogenic waves.

For a better understanding of the results, we compared the accuracy measures of the assessed methods in different clinical conditions related to ICP increase (plateau waves, CSF infusion tests [18], and TBI [31]). The 95% confidence interval for nICP estimation is around 18 mmHg during plateau waves and 15 mmHg in infusion studies, while in TBI it is remarkably smaller, 9.4 mmHg. The black-box model is the best one across these three clinical scenarios. The correlation coefficient in time domain between real and estimated ICP is greatest during plateau waves (around 0.8, except for nICP_FVd [0.63]). For infusion studies, the correlation is much lower (between 0.3 and 0.4 for nICP_BB, FVd, and CrCP). In TBI, the correlation is between 0.5 to 0.6, but only for black-box and PI-based methods.

In respect to ICP accuracy, it is known that even the standard invasive techniques might not present the specified limits for error [32–34], particularly using intraparenchymal microtransducers. Thus, it is debatable whether these accuracy requirements are realistic for all sorts of ICP monitoring. In view of this, an important concept that should be stressed is ICP not solely “as a number,” once dynamical features of this parameter, such as its waveform and relative changes in time, are fundamental for a proper assessment of the clinical state of the patient [35].

The nICP black-box model had been previously analyzed during plateau waves of ICP [20]. In that study, clinical material from 17 patients was used to construct the ICP simulation model, which produced better accuracy and better correlation between real and estimated values of ICP in comparison with our present study. This discrepancy could be associated with the way nICP_BB was generated in both studies. In Schmidt et al.’s previous study [20], the formation dataset was much more specific for plateau waves, in which 7 patients (41.18% of the dataset) presented such phenomena. On the other hand, the formation dataset used for the present study consisted of a large general TBI cohort (n = 140 patients), as mentioned previously.

In this regard, we could generally infer that nICP methods based on formation datasets specific for certain conditions may present better prediction performances than those based on general datasets. An example supporting this inference can be found in another study [31], which demonstrated that the same methods used here presented smaller 95% CI when analyzing a TBI cohort convergent to the formation datasets, i.e., general in terms of different sources of ICP changes. However, the obvious disadvantage of specific formation datasets is the restriction of applicability of the nICP method. This effect was observed when TBI dataset-generated nICP procedures were used for ICP assessment in an NPH cohort subjected to CSF infusion tests [19]. In that study, the use of non-specific nICP formation datasets resulted in rather large deviations from ICP.

Although the evaluated methods generally presented low correlations considering the magnitude of changes in ICP as an absolute value, not all clinical situations involving ICP monitoring may require high accuracy. Alternatively, TCD allows a noninvasive assessment of cerebral circulation dynamics as ICP changes in time domain, with the possibility to track nICP changes in real time in a variety of clinical settings (emergency rooms, ambulatories, operating theatres). This ability is one of the gains of using TCD for nICP monitoring, and it could potentially be applied as an alternative assessment or screening tool where ICP measurements are not part of a standard clinical protocol, or in situations which invasive ICP monitoring is contraindicated.

Plateau waves affect approximately 25% of TBI patients [10] and might also occur in other conditions such as subarachnoid hemorrhage [7], brain tumors, hydrocephalus [8], and craniosynostosis [9]. The identification of these phenomena could represent an important application of TCD-based noninvasive ICP methods for the conditions aforementioned, as plateau waves have been also reported to be associated with poor outcome in TBI patients, especially in regard to their duration [10]. Thus, identifying plateau waves as nICP changes in time domain could eventually provide to the clinician a better understanding of the clinical state of the patient and guide oriented treatments in situations of sustained plateau waves.

### Limitations

The use of radial artery ABP zeroed at the level of the heart instead of the blood pressure in the intracranial compartment can be considered a limitation to this study. This condition might non-accurately approximate peripheral to intracranial ABP, which can specifically change the accuracy of methods relying on ABP waveform analysis. However, heart level zeroing was part of the clinical protocol and could not be changed during the TCD recordings. In addition, changes in cerebrovascular resistance produced by variations in PaCO_2_ may disturb CPP estimation (nCPP) and could act as a confounding factor as this parameter was unavailable.

## Conclusion

The methods of nICP assessment are remarkably accurate in detecting relative changes in ICP during plateau waves across time. Furthermore, they presented high performance in ruling out intracranial hypertension. These characteristics encourage the application of TCD-based nICP in clinical conditions where the knowledge of absolute values of ICP would not be essentially relevant. However, we could infer that the estimation of ICP absolute values by the nICP methods tested is still limited at the current state of development. Such inaccuracy might be related to the matter of non-specific nICP calibration.
